# Acute carbon monoxide poisoning with severe cardiopulmonary compromise: a case report

**DOI:** 10.1186/1757-1626-2-52

**Published:** 2009-01-14

**Authors:** Chang-Teng Wu, Jing-Long Huang, Shao-Hsuan Hsia

**Affiliations:** 1 Department of Pediatrics, Children Medical Center, Chang Gung Memorial Hospital, Chang Gung University College of Medicine, Tauyuan, Taiwan

## Abstract

**Introduction:**

Carbon monoxide (CO) is a colorless, odorless gas produced as a by-product of incomplete combustion, and a common source is the fuel used for heating water in homes. The clinical presentation of CO poisoning may be mild, moderate, or severe.

**Case presentation:**

This paper describes carbon monoxide (CO) poisoning in a 15-year-old child who suffered from severe cardiopulmonary compromise without overt neuropsychiatric sequelae. This occurred after he was exposed to a home heater producing high levels of carbon monoxide for an estimated six to eight hours. CO-induced cardiopulmonary compromise is infrequent in children.

**Conclusion:**

In this case, prolonged exposure to CO resulted in a high carboxyhemoglobin (COHb) concentration, but the child recovered without overt sequelae, despite severe cardiopulmonary compromise.

## Introduction

Carbon monoxide (CO) is a colorless, odorless gas produced as a by-product of incomplete combustion. The clinical presentation of CO poisoning may be mild, moderate, or severe. The most frequently documented complications are persistent or delayed neuropsychiatric sequelae, including dementia, amnestic syndromes, psychosis, and Parkinsonism. These symptoms may be preceded by a lucid period of 2–40 days after the initial exposure [1]. The heart is extremely susceptible to CO-induced hypoxia, due to its high oxygen demand. Cardiac involvement manifests mainly as ischemic insult, with elevated enzyme levels and ECG changes ranging from ST-segment depression to transmural infarction. Conduction abnormalities, atrial fibrillation, prolonged QT interval [2] and ventricular arrhythmia have been demonstrated. There have been few studies of CO-induced cardiopulmonary compromise in children. We report a case of severe cardiopulmonary compromise without overt neuropsychiatric sequelae in a 15-year-old boy. Since such cases are rare in the pediatric literature, the clinical presentation and management are discussed here.

## Case presentation

This 15-year-old boy was found at home with reduced consciousness at about 6:30 am on 25 June 2008 According to his parents' statement, he had slept in his room, which is next to the room containing the home water heater. His father had taken a bath at 11:00 p.m. He was first sent to a local hospital, where he was found to be comatose with urine incontinence and cyanosis. Arterial blood gases showed pH 7.3, PaCO_2 _31.8, PaO_2 _40.9 mmHg, HCO_3 _16, SaO_2 _72%, and SBE -8.5. Hypoxemia was found. His spontaneous breathing was shallow and weak with O2 mask with oxygen 6 L/min. He was intubated and supplied 100% O_2_. Hypotension was detected after intubation. Dopamine 20 μg/kg/min was given. Brain computed tomography revealed brain edema, and the chest x-ray showed bilateral pulmonary edema. The arterial carboxyhemoglobin (COHb) level within 1 hour after discovery was 51.9%. Pertinent laboratory values included ammonia 103 μg/dL, Na 141 mEq/L, K 3.8 mEq/L, Ca 9.9 mg/dL, glucose 198 mg/dL, WBC 17,490, Hb 16.3 g/dL, and platelets 336,000. Fresh blood and pink foamy sputum were noted from the endotracheal tube. Given his critical condition, he was transferred to our hospital for further management.

**Figure 1:**
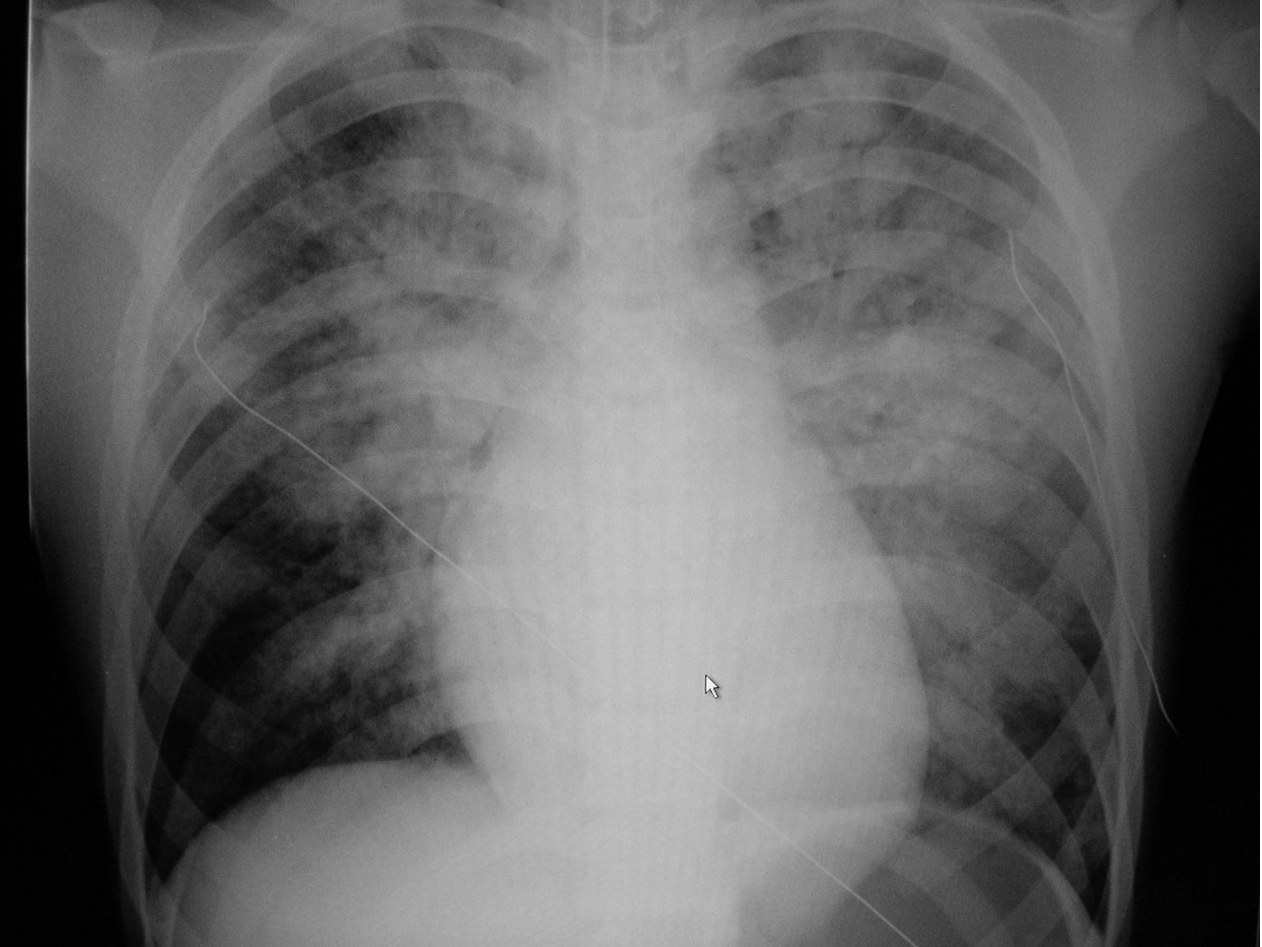
**Chest x-ray showed bilateral acute pulmonary edema**.

One hour after admission, hypotension necessitated the initiation of a dopamine infusion. The infusion was titrated to effect, with 10 μg/kg/min of dopamine. Echocardiography showed an ejection fraction of 55%. Four hours after admission, the arterial COHb was 8.4% and the serum cardiac troponin I was 4.19 ng/mL (normal < 0.4 ng/mL). Acute pulmonary edema was diagnosed by chest x-ray (fig 1). The pulmonary edema was treated with 100% oxygen, with increased positive end-expiratory pressure up to 12 cm H_2_O. The follow-up chest x-ray showed worsening pulmonary edema with increasing bilateral alveolar consolidation. Arterial blood gases showed severe hypoxemia. Acute respiratory distress syndrome was suspected. High-frequency oscillator ventilation was set to maintain a PaO_2 _greater than 50 mm Hg and an oxygen saturation greater than 85%. Beginning 10–12 hours after admission, the pulmonary condition improved progressively. The blood pressure also became more stable (> 90/50 mmHg).

Over the next few days, the patient's cardiovascular and respiratory status kept improving. The serum cardiac troponin I level declined. The neurological findings remained a concern. On day four, the dopamine was discontinued. He was extubated successfully on day five. The neurological findings were normal after extubation. He was transferred from the pediatric intensive care unit to the pediatric ward on day 6, and no longer required oxygen.

## Discussion

In this report, a 15-year-old boy developed cardiopulmonary compromise following CO poisoning. CO intoxication has been properly named "the great imitator" as a comment on the various symptoms with which the victim might present [3]. Common manifestations of acute CO poisoning in children include gastrointestinal symptoms and neurological changes [4-6]. The clinical presentation may mimic a flu-like syndrome or acute gastroenteritis leading to misdiagnosis [7]. Clarke et al described a possible early sign of CO poisoning, euphoria [8].

Carbon monoxide binds to hemoglobin reversibly with an affinity approximately 240 times greater than that of oxygen, thereby reducing the total oxygen-carrying capacity of the hemoglobin. This competitive binding shifts the oxygen-hemoglobin dissociation curve to the left, resulting in the impaired release of oxygen at the tissue level and cellular hypoxia [9]. CO binds to myocardial myoglobin more slowly than it does to hemoglobin, but the bond is stronger and the release slower [10].

The arterial blood gases of this patient showed pH 7.3, PaCO_2 _31.8, PaO_2 _40.9 mmHg, HCO_3 _16, SaO_2 _72%, and SBE -8.5. Hypoxemia was found. But Moyle claimed that the use of pulse oximetry in CO poisoning was negligent. It must now be considered negligent to use pulse oximetry on patients who have been at risk of carbon monoxide inhalation. For every 1% of carboxyhaemoglobin circulating, the pulse oximeter over-reads by approximately 1% [11].

The cardiovascular effects of CO include myocardial ischemia, pulmonary edema, arrhythmia, and stunned myocardium syndrome [12]. Our case had compromised cardiac output and pulmonary edema. This clinical presentation is consistent with the known cardiac effects of carbon monoxide poisoning. Pulmonary edema is uncommon in children with acute CO poisoning. The possible causes of pulmonary edema include toxic effects of CO on the alveolar membranes, myocardial damage leading to left ventricular failure, the aspiration of gastric contents after loss of consciousness, and neurogenic pulmonary edema [13]. The cause of pulmonary edema in our case might have been heart failure resulting from myocardial damage. Our patient recovered successfully with aggressive cardiopulmonary management, including inotropic agents and ventilator support.

This case report is evidence that CO exposure with a high COHb concentration can cause cardiopulmonary compromise in a child; the child was able to recover smoothly, without cerebral failure. The COHb concentrations do not always determine the severity of toxic damage at the level of selected organs, or serve as a prognostic index [14]. Careful clinical evaluation of the cardiopulmonary and neurological systems is advisable in children with CO poisoning.

## Consent

Written informed consent was obtained from the patient for publication of this case report and accompanying images. A copy of the written consent is available for review by the Editor-in-Chief of this journal.

## Competing interests

The authors declare that they have no competing interests.

## Authors' contributions

CTW wrote the manuscript and performed the literature search. JLH and SHH reviewed the manuscript for intellectual content.
